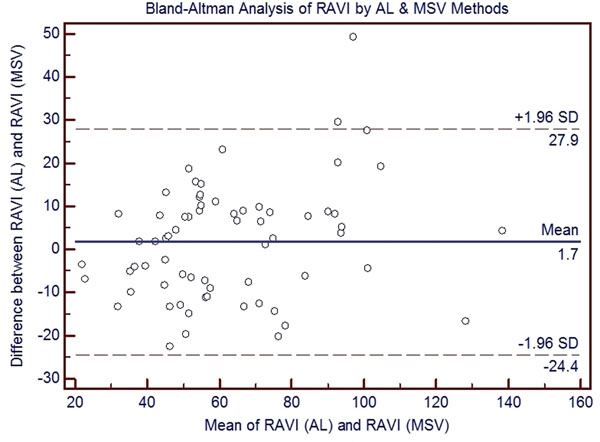# Comparison of two methods of right atrial volume assessment using cardiac magnetic resonance imaging: area-length method versus multislice volumetric method

**DOI:** 10.1186/1532-429X-15-S1-E23

**Published:** 2013-01-30

**Authors:** Santanu Biswas, Thriveni Sanagala, David Wilber, Mushabbar A Syed

**Affiliations:** 1 Medicine, Loyola University Medical Center, Maywood, IL, USA

## Background

Right atrial (RA) volume evaluation is not as well characterized as left atrial (LA) volume assessment. RA volumes are most accurately assessed by the multislice volumetric (MSV) method; however, this method is time consuming requiring manual tracing of RA endocardial borders over several slices. The area-length (AL) method can also be used to estimate RA volume. Although well established for the assessment of LA size, the application of the AL method to the RA is not as well established. We sought to determine the accuracy, limits of agreement, inter-observer, and intra-observer variability of the AL method compared to the MSV method on the measurement of RA volume.

## Methods

We prospectively studied 67 patients who underwent cardiac magnetic resonance imaging (CMR) for a clinically indicated reason. CMR images were acquired on 3 Tesla (Siemens Trio) or 1.5 Tesla (Siemens Aera) scanner using cine steady-state free precession (SSFP) sequence. RA volumes using MSV method were measured from a stack of long-axis slices acquired parallel to the left ventricular 2 chamber plane, which spanned the entire RA and right ventricle (RV). RA volumes using the AL method were measured from a 4-chamber and 2-chamber view of the RA and RV. The RA appendage was included in the RA volumes. RA volumes calculated by MSV and AL methods were compared using Pearson's correlation, regression analysis and Bland-Altman analysis. Inter-observer and intra-observer variability was assessed on a random sample of 10 patients and analyzed by intra-class correlation coefficients and Bland-Altman analysis.

## Results

Mean indexed RA volumes (RAVI) by AL method did not differ significantly when compared with the MSV method (63.7 ± 26.2 vs 62.1 ± 22.7 ml/m^2^, p = 0.29). The AL method correlated highly with MSV method (r = 0.86); however, Bland-Altman analysis demonstrated wide limits of agreement (mean difference 1.7 ± 13.4 ml/m^2^). Intra-observer analysis revealed excellent agreement for both methods (ICC agreement = 0.99 and 0.96, respectively). Inter-observer analysis showed excellent agreement for the MSV method (ICC agreement = 0.95) and strong agreement for the AL method (ICC agreement = 0.76); however, Bland-Altman analysis showed wide limits of agreement.

## Conclusions

RA volumes calculated by AL method correlate well with volumes assessed by MSV method; however, the limits of agreement are wide such that values may not be interchanged between studies. In addition, reliability was superior with the MSV method, which suggests that MSV method may be preferred for serial assessment of RA volumes.

## Funding

None

**Table 1:** Summary Statistics

AL Method versus MSV Method	
Pearson's Correlation for RAVI: AL vs MSV	0.86

Bland-Altman analysis: RAVI: AL vs MSV	1.7 ± 13.4 ml/m^2^

Intra-observer Analysis	

ICC agreement: RAVI (AL)	0.99

ICC consistency: RAVI (AL)	0.99

ICC agreement: RAVI (MSV)	0.96

ICC consistency: RAVI (MSV)	0.96

Bland-Altman analysis: RAVI (AL)	-1.9 ± 5.4 ml/m^2^

Bland-Altman analysis: RAVI (MSV)	2.9 ± 5.0 ml/m^2^

Inter-observer Analysis	

ICC agreement: RAVI (AL)	0.76

ICC consistency: RAVI (AL)	0.73

ICC agreement: RAVI (MSV)	0.95

ICC consistency: RAVI (MSV)	0.95

Bland-Altman analysis: RAVI (AL)	9.2 ± 17.0 ml/m^2^

Bland-Altman analysis: RAVI (MSV)	1.8 ± 5.7 ml/m^2^

**Figure 1:**